# The Contribution of Geography to Disparities in Preventable Hospitalisations between Indigenous and Non-Indigenous Australians

**DOI:** 10.1371/journal.pone.0097892

**Published:** 2014-05-23

**Authors:** Timothy C. Harrold, Deborah A. Randall, Michael O. Falster, Sanja Lujic, Louisa R. Jorm

**Affiliations:** Centre for Health Research, University of Western Sydney, Sydney, New South Wales, Australia; Kyushu University Faculty of Medical Science, Japan

## Abstract

**Objectives:**

To quantify the independent roles of geography and Indigenous status in explaining disparities in Potentially Preventable Hospital (PPH) admissions between Indigenous and non-Indigenous Australians.

**Design, setting and participants:**

Analysis of linked hospital admission data for New South Wales (NSW), Australia, for the period July 1 2003 to June 30 2008.

**Main outcome measures:**

Age-standardised admission rates, and rate ratios adjusted for age, sex and Statistical Local Area (SLA) of residence using multilevel models.

**Results:**

PPH diagnoses accounted for 987,604 admissions in NSW over the study period, of which 3.7% were for Indigenous people. The age-standardised PPH admission rate was 76.5 and 27.3 per 1,000 for Indigenous and non-Indigenous people respectively. PPH admission rates in Indigenous people were 2.16 times higher than in non-Indigenous people of the same age group and sex who lived in the same SLA. The largest disparities in PPH admission rates were seen for diabetes complications, chronic obstructive pulmonary disease and rheumatic heart disease. Both rates of PPH admission in Indigenous people, and the disparity in rates between Indigenous than non-Indigenous people, varied significantly by SLA, with greater disparities seen in regional and remote areas than in major cities.

**Conclusions:**

Higher rates of PPH admission among Indigenous people are not simply a function of their greater likelihood of living in rural and remote areas. The very considerable geographic variation in the disparity in rates of PPH admission between Indigenous and non-Indigenous people indicates that there is potential to reduce unwarranted variation by characterising outlying areas which contribute the most to this disparity.

## Introduction

There is overwhelming evidence that Indigenous Australians, like indigenous peoples worldwide, suffer profound health disadvantage. The life expectancy of Indigenous Australians at birth is around 11.5 years lower for males and 9.7 years lower for females, compared with non-Indigenous Australians [1]. Much of the gap between Indigenous and non-Indigenous Australians is driven by cardiovascular disease, diabetes and related complications, such as renal failure [2]. Similar ethnic disparities for these conditions have been observed in other countries, such as New Zealand [3], the United States of America [4]–[7] and Canada [8].

The concept of the “potentially preventable hospitalisation” (PPH) provides policymakers and health care managers with a framework to identify admissions that may have been prevented if timely and adequate care was available to that individual outside of the hospital system [9]. PPHs are identified using a set of admission diagnosis and procedure codes and are broadly grouped into acute, chronic or vaccine preventable PPH. Rates of PPH admissions, by Indigenous status, are reported by both state and federal governments [10]–[14] and are a key performance indicator specified in the National Healthcare Agreement (NHA), with the aim of reducing PPH admissions to 8.5% of total admissions by 2014–15 [15]. This routine reporting consistently shows that age-adjusted rates of PPH admission are much higher in Indigenous than non-Indigenous Australians, but the magnitude of the differential varies with jurisdiction, from about three-fold in New South Wales (NSW) [11] and Queensland [10] to four-fold in the Northern Territory [9]. Much higher rates of PPH admission are also reported among residents of rural and remote areas [14]. Because Indigenous people make up a greater proportion of the population in rural areas, where admission rates tend to be higher, it is possible that some of the disparity is driven by the differential distribution of the Indigenous population.

Further, evidence from other countries indicates that ethnicity and rurality both contribute to disparities in health care, with rural ethnic minorities experiencing poorer access to health care [16]. Importantly, existing analyses have not quantified the independent roles of geography and Indigenous status in explaining differences in rates of PPH admission between Indigenous and non-Indigenous Australians. Nor have they explored how much this disparity varies among local areas, an essential step in identifying strategies to reduce unwarranted variation.

Our study aimed to address these knowledge gaps.

## Methods

### Ethics approval

Approval for the study was given by the NSW Population and Health Services Research Ethics Committee, the Aboriginal Health and Medical Research Council of NSW Ethics Committee, and the University of Western Sydney Human Research Ethics Committee.

### Study design

Observational study using linked hospital admission records.

### Population

New South Wales (NSW) is the largest state in the Commonwealth of Australia, with a population of 6,663,402 in 2006, and includes both highly urbanised and rural areas. While Indigenous Australians represent only 2.3% of the total population of NSW, 23% of all Indigenous people living in Australia reside in NSW [17]. NSW is comprised of 199 Statistical Local Areas (SLAs) with an average population 35,906 people (range: 364–141,686) and an average spatial area 4,027 km^2^ (range: 4–93,284 km^2^) [18]. SLA was the finest level of geography at which population estimates were available for the study period [18].

### Data

The NSW Admitted Patients Data Collection (APDC) includes information about all separations (discharges, transfers and deaths) from NSW public and private hospitals and day procedure centres. Diagnoses are coded using the International Classification of Diseases and Related Problems, Australian Modification (ICD-10-AM), and procedures are coded using the Australian Classification of Health Interventions Sixth Edition [19].

APDC data were available for the period 1 July 2003 to 30 June 2008. As NSW did not have a unique patient identifier available during the study period, hospital separations associated with the same individual were identified using probabilistic methods by the NSW Centre for Health Record Linkage [20]. Probabilistic matching was performed by the Centre for Health Record Linkage using key personal identifier variables, such as date of birth, first name, last name, sex and residential address to form probability weights for the likelihood of a particular hospital admission being associated with one person. The Centre for Health Record Linkage uses the software package ChoiceMaker, which can adjust for data entry errors, incomplete and missing data [21]. In addition to automated linkage, the CHeReL also conducts a manual clerical review on a sample of records in order to audit linkage quality. False positive and false negative rates for data linkage are 0.4% and less than 0.1%, respectively.

Indigenous people are known to be under-identified in the APDC [22], however there is evidence to indicate that the level of identification improved during the time period of this study [23], [24]. Hence, taking this information into account, an admission was reported as being for an Indigenous person on the basis of the status recorded on their most recent hospital record. This approach increased the number of PPH admissions reported as Indigenous by 5.4%.

We derived synthetic Indigenous population estimates for NSW SLAs using a combination of age- and sex-specific estimates of the total population for SLAs, age- and sex-specific estimates of the Indigenous population for NSW, and the estimated proportion of the population of each SLA that was Indigenous, from the 2006 Australian Census [25], [26]. We estimated the non-Indigenous population by subtracting Indigenous population estimates from the total population.

We identified PPH admissions according to the 2012 NHA performance indicator: Selected potentially preventable hospitalisations [27]. We aggregated the number of PPH admissions by broad PPH grouping and specific condition by strata based on financial year, 10-year age-group (from 0–9 to 80+), sex, Indigenous status and SLA, and then combined these with the estimated population counts. SLAs were grouped into four remoteness categories (major cities, inner regional, outer regional, remote) based on their average Accessibility/Remoteness Index of Australia Plus (ARIA+) score in 2006 [28]. The ARIA+ score measures the remoteness of a point based on the physical road distance to the nearest urban centre in each of five size classes, with the score ranging from 0 (highly accessible) to 15 (high remoteness). ARIA scores are spatially interpolated for a range of different geographical units in order to provide scores for a geographical area. The index excludes socio-economic factors from its calculation [28].

### Statistical analysis

We calculated directly age-standardised admission rates for all PPH conditions and the broad PPH groupings, using events from the hospital data as the numerator and population from the synthetic population estimates as the denominator. The rates were standardised to the 2001 Australian Standard Population [29]. We also calculated average length of stay (ALOS), with the numerator the total number of bed days for each PPH admission and the denominator the total number of PPH admissions.

We used multilevel Poisson models to compare admission rates between Indigenous and non-Indigenous people. All models were adjusted for age group and sex, with the exception of the pelvic inflammatory disease condition-specific model which was only adjusted for age-group, and all models included a random intercept for the SLA of residence. Variation at the SLA level (τ^2^) was expressed as a median rate ratio, which was the median of the rate ratios of pair-wise comparisons of people with identical characteristics taken from randomly chosen SLAs. We also added a random slope for Indigenous status to see whether there was significant variation across areas in the Indigenous to non-Indigenous admission rate ratio. Using area-level “shrunken” residuals from the multilevel models that borrow information from the average to stabilise estimates [30], we estimated PPH admission rates by Indigenous status, and the rate ratio of Indigenous to non-Indigenous admissions, in each SLA. All models included the log of the population as an offset. Strata with no people were excluded.

We used negative binomial multilevel models to compare differences in ALOS between Indigenous and non- Indigenous people. All models were adjusted for age group and sex and included a random intercept for SLA of residence and the log of the number of admissions as an offset. Strata with no admissions were excluded.

Model outputs included adjusted rate ratios (aRR) with their 95% confidence intervals (CI). All analyses were carried out using SAS 9.3 [31] and MLwiN 2.25 [32].

## Results

Over the 5-year study period, PPH diagnoses accounted for 987,604 admissions in NSW. Of these, 36,430 (3.7%) were for Indigenous people. The majority of admissions were for chronic conditions (57%), followed by acute (41%) and vaccine preventable conditions (2.4%), with this distribution being similar for Indigenous and non-Indigenous people (Table 1).

**Table 1:** Number of admissions and age-standardised admission rate by Indigenous status, NSW, 2003/04 to 2007/08.

	Indigenous				Non-Indigenous			
	n	%	ASR	(95% CI)	n	%	ASR	(95% CI)
*Acute conditions*	15044	41.30%	21.97	(21.51–22.43)	385300	40.50%	11.4	(11.37–11.44)
Appendicitis with generalised peritonitis	153	0.40%	0.17	(0.14–0.20)	4938	0.50%	0.15	(0.15–0.15)
Cellulitis	1870	5.10%	3.04	(2.86–3.22)	51459	5.40%	1.48	(1.47–1.49)
Convulsions and epilepsy	3777	10.40%	5.57	(5.36–5.78)	51346	5.40%	1.56	(1.55–1.58)
Dental conditions	2545	7.00%	2.58	(2.46–2.69)	67049	7.00%	2.06	(2.05–2.08)
Ear Nose and Throat conditions	2933	8.10%	2.84	(2.71–2.97)	50118	5.30%	1.56	(1.55–1.58)
Gangrene	116	0.30%	0.29	(0.23–0.36)	5067	0.50%	0.14	(0.14–0.15)
Dehydration and gastroenteritis	1281	3.50%	2.59	(2.41–2.77)	69784	7.30%	2.01	(2.00–2.03)
Pelvic inflammatory disease	284	0.80%	0.39	(0.35–0.44)	7489	0.80%	0.46	(0.45–0.47)
Perforated/bleeding ulcers	158	0.40%	0.4	(0.33–0.48)	8037	0.80%	0.22	(0.22–0.23)
Pyelonephritis	1930	5.30%	4.12	(3.87–4.37)	70191	7.40%	1.99	(1.98–2.00)
*Chronic conditions*	20688	56.80%	53.5	(52.65–54.36)	546973	57.50%	15.38	(15.34–15.42)
Angina	2018	5.50%	5.49	(5.22–5.77)	97683	10.30%	2.73	(2.71–2.74)
Asthma	3062	8.40%	3.77	(3.60–3.93)	60097	6.30%	1.87	(1.85–1.88)
Congestive cardiac failure	1078	3.00%	4.01	(3.73–4.30)	67257	7.10%	1.81	(1.79–1.82)
COPD	3125	8.60%	10.65	(10.23–11.08)	91535	9.60%	2.52	(2.50–2.54)
Diabetes complications	11824	32.50%	31.5	(30.86–32.14)	228072	24.00%	6.38	(6.35–6.40)
Hypertension	318	0.90%	0.79	(0.69–0.90)	10554	1.10%	0.29	(0.29–0.30)
Iron deficiency anaemia	455	1.20%	1.24	(1.11–1.38)	30461	3.20%	0.86	(0.85–0.87)
Nutritional deficiencies	5	0.00%	0.01	(0.00–0.01)	192	0.00%	0.01	(0.00–0.01)
Rheumatic heart disease	124	0.30%	0.2	(0.16–0.25)	2851	0.30%	0.08	(0.08–0.08)
*Vaccine preventable conditions*	882	2.40%	1.51	(1.39–1.65)	23040	2.40%	0.67	(0.66–0.68)
Influenza and pneumonia	682	1.90%	1.22	(1.10–1.34)	18429	1.90%	0.53	(0.53–0.54)
Other vaccine-preventable	201	0.60%	0.3	(0.26–0.35)	4635	0.50%	0.14	(0.13–0.14)
**Overall**	36430	100%	76.48	(75.51–77.46)	951174	100%	27.34	(27.29–27.40)

Note: ASR, age standardised rate per 1000 population; CI, confidence interval. With the exception of pelvic inflammatory disease and nutritional deficiencies, all rates were statistically significantly higher for Indigenous Australians (p<0.05).

The overall age-standardised rate of PPH admissions for Indigenous people was 76.5 per 1 000, compared with 27.3 for non-Indigenous people (Table 1), a ratio of 2.80. Indigenous people experienced significantly higher age-standardised rates of admission for most PPH conditions, with the exception of appendicitis, pelvic inflammatory disease and nutritional deficiencies (Table 1).

**Figure 1:**
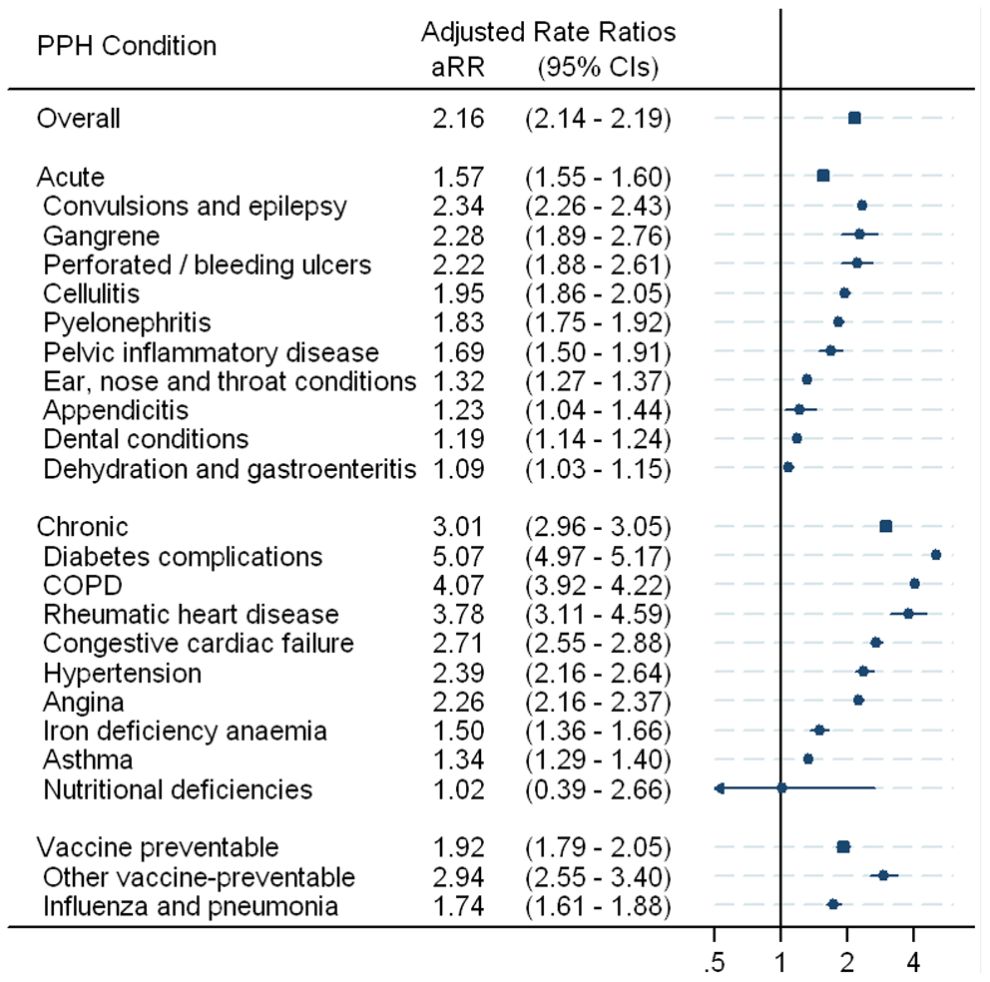
Adjusted admission rate ratio for selected PPH conditions, for Indigenous people compared with non-Indigenous people, 2003/04 to 2007/08, after adjustment for age group, sex and area of residence.

Figure 1 presents Indigenous to non-Indigenous rate ratios, adjusted for age, sex and geographic clustering by including a random intercept for SLA in multilevel models. After adjusting for geographic clustering the magnitude of the Indigenous to non-Indigenous overall PPH aRR decreased from 2.58 (95% CI 2.55–2.60) to 2.16 (95% CI 2.14–2.19), indicating that PPH admission rates in Indigenous people were 2.16 times higher than in non-Indigenous people of the same age group and sex who lived in the same SLA. This indicated that geographic clustering accounted for only some of the observed disparity. Significantly higher rates of PPH admissions for Indigenous people were found for all PPH conditions with the exception of nutritional deficiencies (for which numbers were very small) (Figure 1). The SLA-level variation was equivalent to a median rate ratio of 1.50; in other words, for any population group defined by age, sex and Indigenous status from two randomly chosen areas, PPH admissions in one area were on average 50% higher than in the other area.

After adjusting for age, sex and SLA, the largest disparities in PPH admission rates were seen for diabetes complications (aRR = 5.07, 95% CI 4.97–5.17), COPD (aRR = 4.07, 95% CI 3.92–4.22), rheumatic heart disease (aRR = 3.78, 95% CI 3.11–4.59), other vaccine preventable (aRR = 2.94, 95% CI 2.55–3.40) and congestive cardiac failure (aRR = 2.71, 95% CI 2.55–2.88) (Figure 1).

**Figure 2:**
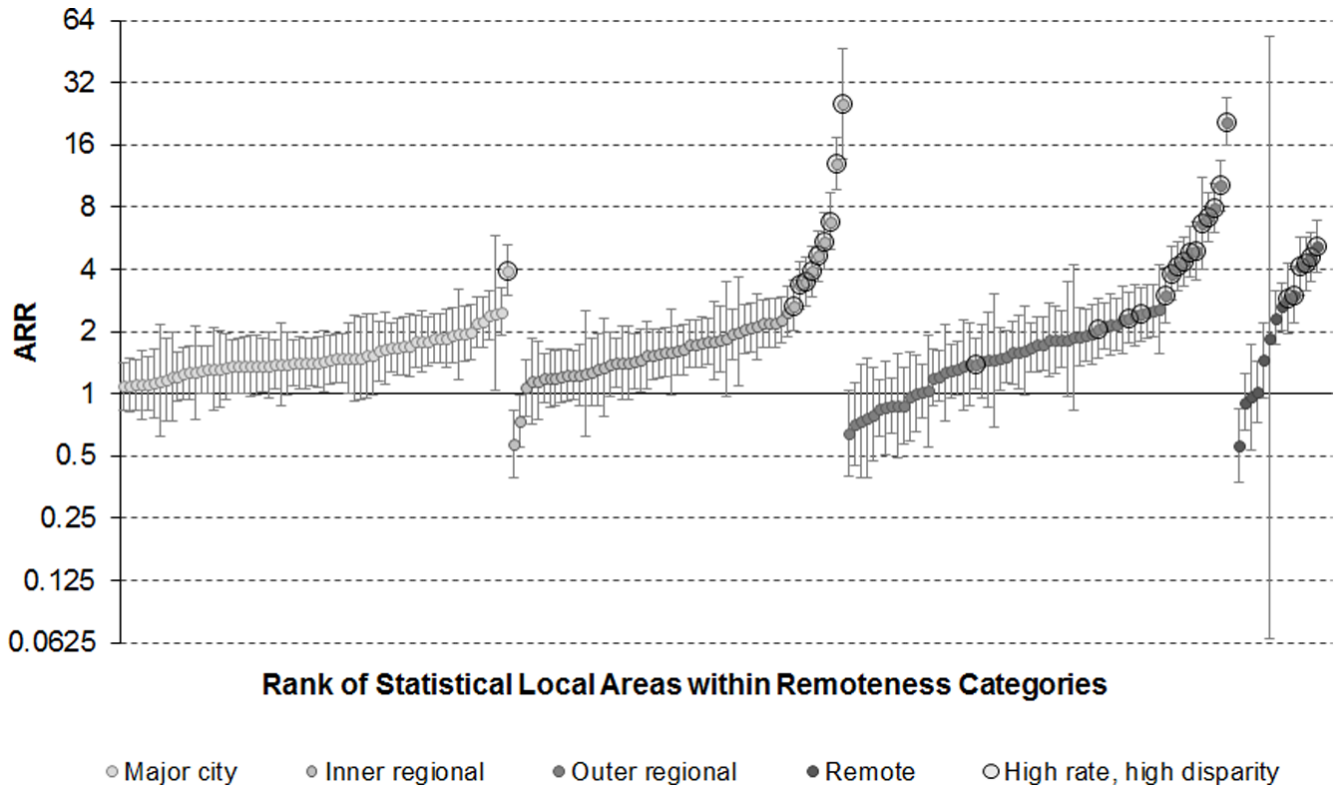
Indigenous to non-Indigenous PPH admission rate ratio by Statistical Local Area and remoteness categories, 2003/04 to 2007/08, adjusted for age group and sex.

Rates of PPH admission varied markedly according to SLA (p<0.001) (from the random intercept model) and the rate ratio of Indigenous to non-Indigenous admissions also varied significantly (p<0.001) (from the random intercept and random slope model). Figure 2 plots the variation in Indigenous to non-Indigenous rates of PPH admission by SLA and remoteness category, and highlights SLAs where the age- and sex-adjusted Indigenous rate of PPH admission was higher than the state average for Indigenous people as well as being higher than the adjusted non-Indigenous rate in that area. Figure S1 plots the variation in Indigenous to non-Indigenous rate ratios of PPH admission by SLA on a map of NSW. It shows that rates of PPH admission were higher in Indigenous than non-Indigenous people in the vast majority of SLAs, with greater disparities seen in regional and remote areas than in major cities. More than 30 SLAs, mainly in regional areas, had both higher than average Indigenous rates of PPH admissions and higher than average disparities in rates between Indigenous and non-Indigenous people. These “high rate, high disparity” SLAs are shown in Table 2. Three SLAs, Hay, Junee and Lithgow (C), had both lower than average Indigenous rates of PPH admissions and lower than average disparities in rates between Indigenous and non-Indigenous people. Both Junee and Lithgow (C) are inner regional areas, while Hay is a remote area according to the ARIA+ remoteness classification.

**Table 2:** “High rate, high disparity” Statistical Local Areas.

Remoteness Area	Statistical Local Areas
Major cities	Sydney - South
Inner regional	Clarence Valley (A) - Grafton, Wagga Wagga - Part A, Wagga Wagga - Part B, Lismore - Part A, Armidale Dumaresq - City, Ballina, Richmond Valley - Balance, Byron, Richmond Valley - Casino
Outer regional	Warrumbungle Shire, Tumut, Griffith, Gilgandra, Narrabri, Tenterfield, Armidale Dumaresq - Balance, Eurobodalla, Kyogle, Inverell - Part B, Broken Hill, Nambucca, Narrandera, Wellington, Bega Valley, Kempsey, Murrumbidgee
Remote/Very Remote	Balranald, Brewarrina, Central Darling, Bourke, Carrathool, Walgett, Lachlan, Coonamble, Warren

Average length of stay for PPH admissions in Indigenous people was slightly longer than for non-Indigenous people of the same age group and sex who lived in the same SLA (aRR 1.05, 95% CI 1.02–1.08), with this difference being statistically significant for acute (aRR 1.08, 95% CI 1.05–1.12) and chronic (aRR 1.04, 95% CI 1.01–1.08) but not vaccine-preventable (aRR 1.00, 95% CI 0.86–1.16) conditions. Including a random slope term for Indigenous status did not markedly alter our estimates of the disparity in Indigenous and non-Indigenous average length of stay (not shown).

## Discussion

Ours was the first study to our knowledge to explore the independent roles of geography and Indigenous status in explaining differences in rates of PPH admission between Indigenous and non-Indigenous people. Our results demonstrated unequivocally that higher rates of PPH admission among Indigenous people are not simply a function of their greater likelihood of living in rural and remote areas where rates of PPH admissions are higher [14]. The slightly longer length of PPH hospital stays for Indigenous than non-Indigenous patients who lived in the same SLA suggested that it was unlikely that a “lower threshold” for admission of Indigenous patients was a major contributor to the observed disparities. However, longer stays could reflect lesser availability of assistance with care at home or in the community, as well as greater disease severity. Further, it is possible that longer stays may also reflect differences in hospital discharge practices or the types of hospitals that Indigenous and non-Indigenous people seek treatment from.

Our age-standardised rates of hospitalisation for PPH diagnoses in Indigenous people in NSW (76.5 admissions per 1,000) were lower than those reported for Indigenous people in the Northern Territory (110 per 1,000) in the years 1998–99 to 2005–06 [9], while the rates for non-Indigenous people were similar in both studies (27.3 and 27.8 per 1,000 respectively). Likely explanations include higher incidence and prevalence of PPH conditions in the NT Indigenous population, differences in the prevalence of behavioural risk factors that contribute to the risk of developing specific PPH conditions, differences between the jurisdictions in the provision and accessibility of primary health care and hospital services, and possibly better identification of Indigenous people in NT hospital data [22]. However, audits of NSW hospital data found that about 88% of admissions were correctly recorded in 2007 and that 91% in 2010 were correctly identified in NSW public hospitals [22], [23]. Also, we enhanced the reporting of Indigenous status by using the most recent hospital record for each individual.

We found that after adjusting for age, sex and SLA of residence, rates of PPH admission in Indigenous people were significantly higher than those in non-Indigenous people across almost all conditions included in the PPH indicator. However, diabetes complications contributed around one-third of all PPH admissions in Indigenous people, and were also responsible for the largest disparity, with the rate of these admissions for Indigenous people being more than five times higher than for non-Indigenous people of the same age group, sex and SLA of residence. Large ethnic disparities in potentially avoidable hospitalisations for diabetes were also evident between New Zealand Māori and people of European descent [3], and African Americans and non-Hispanic Whites in the United States of America (USA) [4], [5]. Our finding reinforces the importance of tackling the determinants of diabetes, and better diabetes management, as key priorities for improving the health of Indigenous Australians.

We found that there was very considerable geographic variation in the disparity in rates of PPH admission between Indigenous and non-Indigenous people in NSW, presenting the potential to reduce disparities by characterising and targeting the sources of this variation. We identified more than 30 “high rate, high disparity” SLAs in NSW, mainly in regional areas, as well as three “low rate, low disparity” SLAs. However, using administrative hospital data alone, we were unable to identify the relative contributions of such factors as differences in underlying disease prevalence, disease severity, access to quality care, and admission practices to these variations in admission rates. While studies of ambulatory care-sensitive hospitalisations in the USA were able to account for underlying disease prevalence in their estimates of ethnic disparities [4], [5], these did not examine ethnic disparities according to small geographical areas.

We could not identify other studies that investigated how ethnic disparities in potentially preventable hospitalisation varied with geography. Findings for disparities in mortality have varied between settings. Studies in New Zealand have reported relatively little variation in disparities between New Zealand Māori and European/other populations in life expectancy [33] and mortality [34] at the District Health Board level. Research in Massachusetts, USA [35] reported substantial variations in disparities in mortality between Black and White populations at the Census Tract level, while this variation was not found using similar methods for the more urbanised population of Los Angeles [36]. These contrasting findings emphasise the importance of methods that are able to account for both the person and their place, such as multilevel modelling, in studies of ethnic disparities in health [16], [34].

A possible artefactual contributor to geographic variation in our study was inconsistency in the numerator (hospital admission) and denominator (population census) data that were used to calculate admission rates. For example, high mobility of Indigenous people [37] between their main rural place of residence and inner Sydney might contribute to the high PPH admission rates observed in Sydney South SLA.

Linkage of hospital data to other population-based data such as large-scale health survey data, disease registers or Medicare claims would go some way towards addressing the limitations of our study, by ensuring consistency between numerator and denominator data, and providing more detailed information about patient risk factors. Unfortunately such linkages are not presently available as part of the current National Aboriginal and Torres Strait Islander Health Survey, and processes for access to linked Medicare and other Commonwealth data for research, while currently being revised [38], are very difficult to navigate.

Although we urgently need more information to characterise the sources of geographic variation in PPH admission rates, evidence is starting to emerge about the types of interventions that might be successful in tackling these variations. These include chronic disease management interventions that place an emphasis on the Chronic Care Model [39], recall and reminder systems for people with diabetes [40], ensuring that Indigenous people have access to culturally appropriate health care services designed to meet their specific needs [41] and the application of continuous quality improvement principles to Indigenous primary health care services, such as in the Healthy for Life program [42].

Repeating our analyses using linked hospital data for the whole of Australia, when available, will allow exploration of inter-jurisdictional differences. It will also open up possibilities for applying novel evaluation methods using “natural experiments” [43] to identify the features of current programs and services that are associated with lower rates of potentially preventable hospitalisation among Indigenous Australians.

## Supporting Information

Figure S1 **Map of Indigenous to non-Indigenous PPH admission rate ratio by Statistical Local Area, 2003/04 to 2007/08, adjusted for age group and sex.**
(TIF)
