# Supplementary figures and images for: The Contribution of Geography to Disparities in Preventable Hospitalisations between Indigenous and Non-Indigenous Australians
Source: PLoS One. 2014 May 23;9(5):e97892. doi: 10.1371/journal.pone.0097892 (PMC4032338; doi:10.1371/journal.pone.0097892)

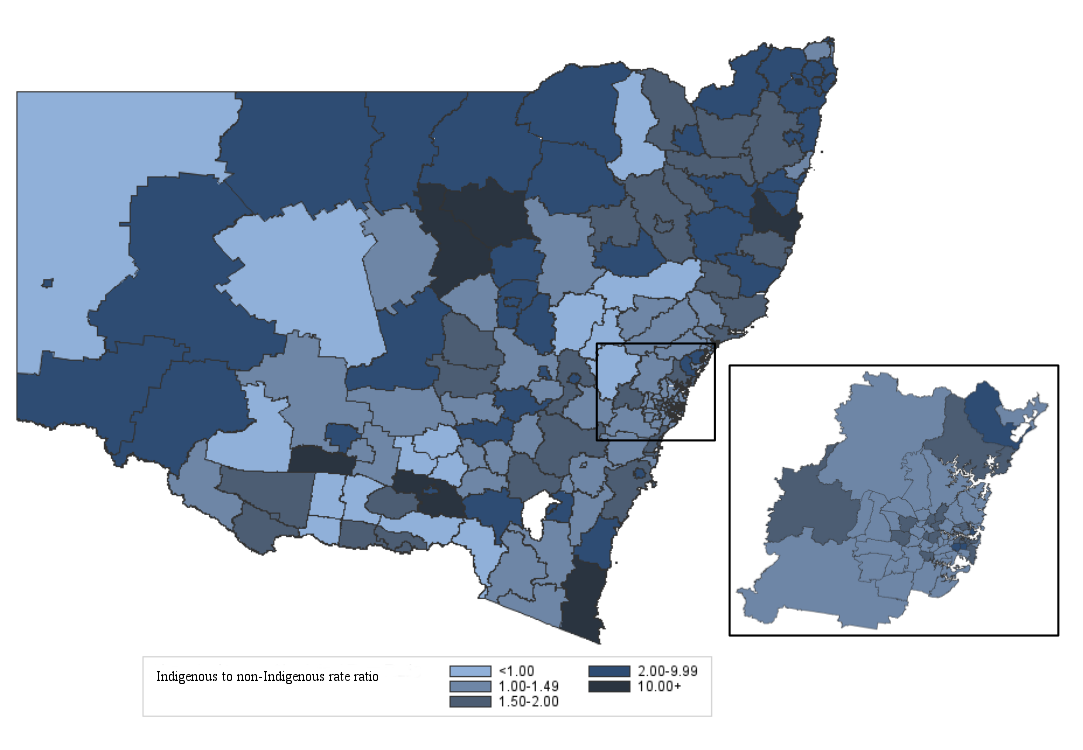

Supplement: Figure S1 — Map of Indigenous to non-Indigenous PPH admission rate ratio by Statistical Local Area, 2003/04 to 2007/08, adjusted for age group and sex. (TIF) [file pone.0097892.s001.tif]
